# Biological and social reproductive factors and late‐life cognitive function in middle‐aged and older Chinese women

**DOI:** 10.1002/alz.70824

**Published:** 2025-10-22

**Authors:** Xueqin Li, Wanyu Zhao, Huirong Zheng, Bangshan Liu, Hongbo He, Jin Liu, Ling Jiang Li, Yan Zhang

**Affiliations:** ^1^ Guangdong Cardiovascular Institute Guangdong Provincial People's Hospital Ganzhou Hospital Guangdong Academy of Medical Sciences Ganzhou Jiangxi China; ^2^ Guangdong Mental Health Center Guangdong Provincial People's Hospital (Guangdong Academy of Medical Sciences) Southern Medical University Guangzhou Guangdong China; ^3^ Center of Gerontology and Geriatrics and National Clinical Research Center of Geriatrics West China Hospital Sichuan University Chengdu Sichuan China; ^4^ The Second Clinical School of Medicine Southern Medical University Guangzhou Guangdong China; ^5^ Department of Psychiatry National Clinical Research Center for Mental Disorders and National Center for Mental Disorders The Second Xiangya Hospital of Central South University Changsha Hunan China

**Keywords:** Chinese middle‐aged and older women, cognitive function, number of children, reproductive lifespan, sex differences

## Abstract

**INTRODUCTION:**

Few studies have concurrently examined the biological and social reproductive factors in relation to women's cognitive aging.

**METHODS:**

We analyzed 8577 women and 7872 men ≥45 years of age from the China Health and Retirement Longitudinal Study. Biological reproductive factors included reproductive span, age at menarche, and age at menopause; social reproductive factors included number of children and age at first live birth. Multivariable regression models were sequentially adjusted for age, childhood cognition proxy, education, and current health and lifestyle factors.

**RESULTS:**

Longer reproductive span was associated with better cognitive performance in women, whereas a higher number of children were linked to poorer cognition in both sexes, particularly in women. These associations remained robust after full adjustment, compared with age at menarche, age at menopause, and age at first birth.

**CONCLUSION:**

Integrating biological and social reproductive factors provides insights into sex‐specific cognitive aging patterns and may inform tailored dementia prevention strategies.

**Highlights:**

- A longer reproductive span was linked to better cognition in older Chinese women.
- More children were linked to poorer cognition in both sexes, especially in women.
- Reproductive span and number of children showed robust associations with late‐life cognition, stronger than other reproductive factors.

## 1 BACKGROUND

Dementia is a growing global health crisis, contributing substantially to morbidity, mortality, and health care costs. According to the World Health Statistics 2024, Alzheimer disease and other dementias rank as the seventh leading cause of death from noncommunicable diseases worldwide and are among the top five in the Americas, Europe, and the Western Pacific Region.^1^ In the United States, 11.3% of adults 65 years of age or older are affected by Alzheimer disease—73% of whom are 75 years of age or older—and this number is projected to double by 2060.^2^ The economic burden is equally alarming, as per capita Medicare expenditures for individuals with dementia are nearly three times higher than for those without, and Medicaid spending is over 22 times greater.^3^

In China, where population aging is accelerating rapidly, dementia poses a major public health concern. An estimated 6.0% of individuals 60 years of age or older have dementia, whereas 15.5% have mild cognitive impairment (MCI).^4^ Among G20 nations, China bears the highest burden of dementia, with a 322% increase in cases and a 273% rise in related health losses over the past three decades, driven primarily by demographic aging.^5^ By the end of 2023, ≈297 million people in China were 60 years or older, comprising 21.1% of the total population.^6^ These trends underscore the need for targeted strategies to delay or prevent cognitive decline.

Women bear a disproportionate burden of dementia globally. In the United States, nearly two‐thirds of patients with Alzheimer's disease are women.^2^ Similarly, in China, dementia affects 7.0% of women versus 5.0% of men; MCI rates are 17.9% versus 13.0%.^4^ These differences persist after adjusting for age, suggesting that factors beyond longevity may contribute.^5^ Emerging evidence highlights the role of reproductive factors in shaping women's cognitive aging. These factors, encompassing both biological and social reproductive dimensions, may jointly influence women's long‐term cognitive health.^7^ Biologically, estrogen is believed to exert neuroprotective effects. Epidemiological studies have linked longer reproductive span,^8^ later age at menopause,^8^, ^9^, ^10^ and prolonged exposure to endogenous estradiol^11^, ^12^ with reduced risk of cognitive decline and Alzheimer's disease. Conversely, accelerated reproductive aging has been linked to adverse cognitive outcomes.^13^ A recent cohort study in China reported that extreme menopausal age—whether premature, early, or late—was associated with poorer cognitive outcomes in later life.^14^ The social dimension of reproduction, commonly measured as number of children, has also been linked to cognitive aging. A higher number of children has been associated with poorer cognitive performance across multiple domains.^15^, ^16^ Moreover, recent research among African Caribbean women has indicated that older age at first live birth was significantly associated with better late‐life cognitive function, suggesting that reproductive timing may influence long‐term cognitive health.^17^

Despite an increasing interest in the intersection of reproductive history and cognitive health, few studies have examined both biological and social reproductive factors in tandem. Moreover, most existing evidence comes from high‐income countries. Few population‐based studies have explored these associations in non–high‐income settings such as China, where social norms around fertility and structural disparities in education and health care may shape these relationships in distinct ways. To address this critical gap, we analyzed longitudinal data from the China Health and Retirement Longitudinal Study (CHARLS) to examine how both biological indicators of ovarian aging (i.e., reproductive span, age at menopause) and social reproductive factors (i.e., number of children) are associated with cognitive function in later life. Drawing on a nationally representative sample of midlife and older adults, we tested the following hypotheses: (1) a longer reproductive span is positively associated with late‐life cognitive performance in women; (2) a higher number of children is negatively associated with late‐life cognition in both sexes, with stronger effects in women. By integrating both biological and social reproductive exposures, this study aims to clarify sex‐specific mechanisms underlying cognitive aging and to inform more equitable and tailored dementia prevention strategies.

## 2 METHODS

### 2.1 Study population

**RESEARCH IN CONTEXT**

1. **Systematic review**: We reviewed the literature using traditional search engines (e.g., PubMed and Google Scholar). Few studies have integrated both the biological and social aspects of ovarian function in relation to cognitive aging in women. Most evidence comes from Western populations, mainly from North America and Europe, with limited data from nationally representative Asian cohorts.
2. **Interpretation**: Using data from more than 8000 Chinese women, this study found that that longer reproductive span was associated with better late‐life cognitive function. A higher number of children was linked to poorer cognition in both sexes, with stronger effects in women. These associations remained robust even after full adjustment, relative to age at menarche, age at menopause, and age at first birth.
3. **Future directions**: Future studies should explore modifiable psychosocial and lifestyle mediators of cognitive aging in diverse populations. Our findings highlight the relevance of reproductive factors in dementia risk assessment and suggest potential intervention targets for women with high parity.

We used data from the China Health and Retirement Longitudinal Study (or CHARLS), a nationally representative longitudinal cohort study of community‐dwelling individuals ≥45 years of age in China. CHARLS collects detailed information on demographic characteristics, health status, economic conditions, and social factors.^18^ For this study, we included data from Wave 1 (2011) through Wave 4 (2018).^19^ Eligible participants were ≥45 years of age, had completed at least one wave of cognitive assessments, and had available data on number of children or reproductive lifespan. A total of 16,449 individuals met these criteria.

**FIGURE 1:**
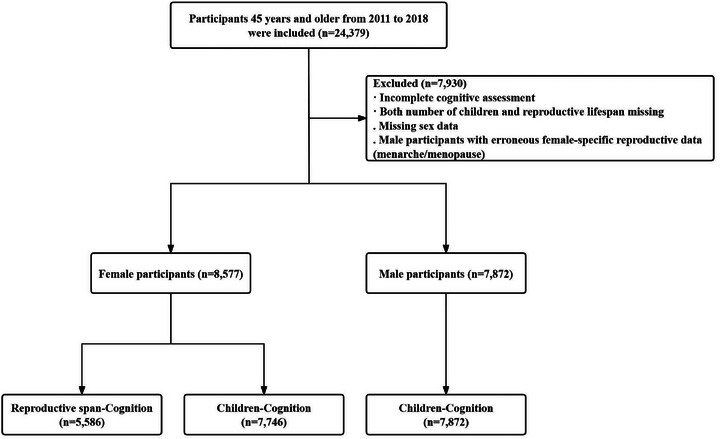
Study flow chart.

Among the eligible participants, 8577 women and 7872 men were included in the analysis of the association between number of children and cognitive function. For the analysis of reproductive lifespan and postmenopausal cognitive function, 5586 postmenopausal women were included in the study (Figure 1). Supplementary analyses involved 6879 women for age at menarche and 6396 women for age at natural menopause. Although a small number of participants (*n* = 117) reported a history of ovarian, cervical, or endometrial cancer, they were retained in the sample because the limited case numbers and missing treatment details precluded meaningful assessment of their impact on reproductive factors.

### 2.2 Measures

#### 2.2.1 Outcome: Cognitive function

Cognitive function was assessed using a composite score derived from neuropsychological tasks adapted from the Health and Retirement Study (HRS),^19^ covering two domains: episodic memory and executive function. Executive function was assessed by tasks measuring orientation (date naming), calculation (serial sevens), and visuospatial ability (figure drawing), with scores ranging from 0 to 11. Episodic memory was evaluated via immediate and delayed word recall (range: 0–10). A global cognition score was computed by summing the two domain scores (range: 0–21), with higher scores indicating better performance. To facilitate interpretation, a global cognition score was standardized into z‐scores using baseline means and standard deviations, with positive values indicating above‐average cognition and negative values indicating below‐average performance. For further analysis, these z‐scores were dichotomized at zero, with scores ≥0 classified as the high cognitive group and scores <0 as the low cognitive group.

#### 2.2.2 Exposure

##### 2.2.2.1 Biological reproductive factors

The primary exposure was reproductive lifespan, defined as the interval between age at menarche and age at natural menopause. Age at menarche and age at natural menopause were obtained via self‐report. When direct age values were unavailable, age was derived from the reported year of menarche or menopause minus the participant's birth year. All implausible values were identified and excluded based on the interquartile range (IQR) method: values below Q1 − 1.5 × IQR or above Q3 + 1.5 × IQR. For further analysis, reproductive lifespan was dichotomized at the sample median, with values ≥ median classified as long and values < median classified as short.

##### 2.2.2.2 Social reproductive factors

Number of biological children was used to represent the social reproductive dimension. Using data from the 2014 CHARLS Life History Survey, participants were categorized into fewer children (1–2 children) and more children (>2 children) groups based on the sample median. Both women and men were included in the analysis to examine sex‐specific associations between number of children and cognitive function. Age at first birth was calculated as the difference between the participant's birth year and the birth year of the first child.

#### 2.2.3 Covariates

Potential confounders were adjusted for by including childhood cognitive proxy variables, as well as sociodemographic, lifestyle, and health‐related characteristics. Due to the lack of direct measures of childhood cognition in the cohort, childhood cognitive proxies included mother's education, father's education, childhood family economic status, childhood family safety, and childhood physical health, all obtained from the 2014 CHARLS Life History Survey. Other covariates were derived primarily from the most recent wave (2018) and supplemented sequentially by 2015, 2013, and 2011, if missing. Educational level was categorized into four groups—preprimary, primary, lower secondary, and upper secondary and above—based on the International Standard Classification of Education (ISCED) 2011^20^ and CHARLS coding. Other covariates included age, sex (male/female), residence type (urban, integration zone, rural, or special zone), marital status (married vs others), smoking status (never, former, or current smoker), and drinking status (never, former, or current drinker). Chronic disease status was also considered, focusing on conditions relevant to women's reproductive health and cognitive function, including hypertension, dyslipidemia, diabetes, heart disease, stroke, psychiatric problems, and memory‐related diseases.^21^, ^22^, ^23^, ^24^ It is important to note that age, which is closely related to late‐life cognitive performance, was taken from the same survey wave as the cognitive assessment to ensure temporal consistency. All variable details are described in full in the Supplementary Methods.

#### 2.2.4 Statistical analysis

All analyses were based on cross‐sectional data from the 2018 wave of CHARLS, with information from earlier waves (2011, 2013, and 2015) used only to supplement missing variables. Descriptive statistics were used to characterize the study population. Continuous variables were presented as means and SDs, whereas categorical variables were summarized as counts and percentages. All variables were examined by stratifying participants according to reproductive lifespan (short vs long) and number of children (fewer vs more). Group differences in sociodemographic, lifestyle, and health‐related variables were assessed using independent two‐sample *t*‐tests for continuous variables and chi‐square tests for categorical variables.

Univariate and multivariable logistic regression models were applied to estimate odds ratios (ORs) and 95% confidence intervals (CIs) for the associations of reproductive lifespan and number of children with cognitive function. Potential confounders were selected based on biological plausibility and previous literature linking these factors with cognitive outcomes. A series of multivariable logistic regression models were constructed using a stepwise adjustment approach. Model 1 adjusted for age and childhood cognitive proxies, including residential location, mother's education, father's education, childhood family economic status, childhood family safety, and childhood physical health. Model 2 additionally included adult education, whereas Model 3 further accounted for marital status, drinking status, smoking status, and physical diseases. Finally, in Model 4, we considered the potential collinearity and overlapping explanatory value between childhood cognitive proxies and adult education, and therefore excluded childhood proxies while retaining adult education to better evaluate its independent contribution to cognitive function.

All analyses were performed using Stata version 18.0 (StataCorp LLC, College Station, TX), with two‐tailed *p* values < 0.05 considered statistically significant.

## 3 RESULTS

### 3.1 Reproductive lifespan and cognition

#### 3.1.1 Characteristics of the study population

Table 1 presents the characteristics of 5586 postmenopausal women, stratified by reproductive lifespan based on the median split. Of these, 2653 (47.5%) had a short reproductive lifespan and 2933 (52.5%) had a long lifespan. The mean age ± SD was 62.4 ± 8.5 years. Women with longer reproductive lifespans were more likely to exhibit high cognitive function (48.8% vs 40.5%) and to have fewer children (1–2 children: 52.5% vs 47.4%). They also reported later menarche (54.7%) and menopause (85.9%), whereas earlier reproductive milestones were more common in the short‐span group (menarche: 77.7%; menopause: 75.8%). In addition, women in the long reproductive span group had higher educational attainment (lower secondary: 19.5% vs 15.7%; upper secondary or above: 13.5% vs 7.8%), were more likely to report urban residence, current marriage, and current alcohol use.

**TABLE 1:** Postmenopausal participant characteristics by reproductive lifespan in the China Health and Retirement Longitudinal Study, 2011–2018.

Characteristic	Overall *n* = 5586	Short span *n* = 2653	Long span *n* = 2933	*p* value
Age, years, mean (SD)	62.4 (8.5)	62.7 (8.7)	62.1 (8.4)	0.013^*^
Education, *n* (%)				<0.001^*^
Preprimary	1623 (29.1)	860 (32.4)	763 (26.0)	
Primary	2368 (42.4)	1169 (44.1)	1199 (40.9)	
Lower secondary	989 (17.7)	416 (15.7)	573 (19.5)	
Upper secondary or above	605 (10.8)	208 (7.8)	397 (13.5)	
Residence, *n* (%)				<0.001^*^
Urban	1092 (21.0)	424 (17.2)	668 (24.5)	
Intergration zone	362 (7.0)	158 (6.4)	204 (7.5)	
Rural	3741 (72.0)	1885 (76.3)	1856 (68.0)	
Special zone	3 (0.1)	2 (0.1)	1 (< 1)	
Mother education, *n* (%)				0.043^*^
Preprimary	4080 (88.2)	1978 (89.4)	2102 (87.1)	
Primary	451 (9.8)	200 (9.0)	251 (10.4)	
Lower secondary	61 (1.3)	24 (1.1)	37 (1.5)	
Upper secondary or above	32 (0.7)	10 (0.5)	22 (0.9)	
Father education, *n* (%)				0.34
Preprimary	2474 (56.4)	1206 (57.6)	1268 (55.3)	
Primary	1503 (34.3)	701 (33.5)	802 (35.0)	
Lower secondary	236 (5.4)	103 (4.9)	133 (5.8)	
Upper secondary or above	173 (3.9)	83 (4.0)	90 (3.9)	
Childhood economy, *n* (%)				0.018^*^
Better	506 (10.6)	213 (9.3)	293 (11.8)	
Same	2459 (51.4)	1187 (51.8)	1272 (51.1)	
Worse	1817 (38.0)	892 (38.9)	925 (37.1)	
Childhood safe, *n* (%)				0.58
Very safe	2334 (50.1)	1129 (50.6)	1205 (49.7)	
Somewhat safe	1914 (41.1)	900 (40.4)	1014 (41.8)	
Not safe	408 (8.8)	201 (9.0)	207 (8.5)	
Childhood health				0.19
Healthier	1665 (34.9)	768 (33.6)	897 (36.1)	
Average	2455 (51.5)	1198 (52.5)	1257 (50.6)	
Less healthy	645 (13.5)	317 (13.9)	328 (13.2)	
Marital status, *n* (%)				0.015^*^
Married	4330 (78.9)	2021 (77.5)	2309 (80.1)	
Others	1160 (21.1)	588 (22.5)	572 (19.9)	
Smoke, *n* (%)				0.96
Never smoke	4410 (92.8)	2091 (92.9)	2319 (92.7)	
Former smoke	119 (2.5)	56 (2.5)	63 (2.5)	
Current smoke	224 (4.7)	104 (4.6)	120 (4.8)	
Drink, *n* (%)				0.016^*^
Never drink	4200 (75.2)	2009 (75.7)	2191 (74.7)	
Former drink	421 (7.5)	219 (8.3)	202 (6.9)	
Current drink	965 (17.3)	425 (16.0)	540 (18.4)	
Disease, *n* (%)				0.37
Yes	3643 (65.5)	1748 (66.1)	1895 (65.0)	
No	1916 (34.5)	895 (33.9)	1021 (35.0)	
Menarche age	16.1 (2.1)	16.9 (1.9)	15.2 (1.8)	<0.001^*^
Menarche age, *n* (%)				<0.001^*^
Early menarche	3352 (60.7)	2037 (77.7)	1315 (45.3)	
Late menarche	2174 (39.3)	584 (22.3)	1590 (54.7)	
Menopause age	49.5 (3.6)	47.0 (3.0)	51.8 (2.4)	<0.001^*^
Menopause age, *n* (%)				<0.001^*^
Early menopause	2399 (43.4)	1989 (75.8)	410 (14.1)	
Late menopause	3131 (56.6)	634 (24.2)	2497 (85.9)	
Reproductive life span	33.4 (4.1)	30.0 (2.8)	36.5 (2.2)	<0.001^*^
Children	2.8 (1.4)	2.9 (1.4)	2.7 (1.4)	<0.001^*^
Children, *n* (%)				<0.001^*^
Fewer children	2381 (50.1)	1083 (47.4)	1298 (52.5)	
More children	2374 (49.9)	1201 (52.6)	1173 (47.5)	
Age at first live birth	24.8 (4.2)	24.7 (4.2)	24.9 (4.1)	0.18
Age at first live birth, *n* (%)				0.013^*^
Early age at first birth	2201 (52.7)	1091 (54.7)	1110 (50.8)	
Late age at first birth	1979 (47.3)	905 (45.3)	1074 (49.2)	
Cognitive z‐score	−0.2 (1.0)	−0.3 (1.0)	−0.1 (1.1)	<0.001^*^
Cognitive function, *n* (%)				<0.001^*^
Low	3082 (55.2)	1579 (59.5)	1503 (51.2)	
High	2504 (44.8)	1074 (40.5)	1430 (48.8)	

* *p* < 0.05.

#### 3.1.2 Association between reproductive lifespan and cognition

Table 2 presents the associations of reproductive lifespan, age at menarche, and age at menopause with cognitive function among postmenopausal women. A longer reproductive lifespan was significantly associated with higher odds of high cognitive function compared with a shorter span (OR = 1.40, 95% CI: 1.26–1.56). This association remained robust after further adjustment for childhood cognitive proxies (OR = 1.31, 95% CI: 1.15–1.50) and adult education (OR = 1.20, 95% CI: 1.03–1.38), and the trend persisted in other adjusted models, including Model 3 (OR = 1.15, 95% CI: 0.99–1.34) and Model 4 (OR = 1.20, 95% CI: 1.05–1.37) (see Table S1). Consistent results were observed in linear regression analyses, further supporting the positive association between reproductive lifespan and late‐life cognition in women (Table S2). In contrast, the associations of later menarche and later menopause with cognitive function appeared less consistent. Later menarche was significantly associated with a higher likelihood of better cognitive performance only in the unadjusted model (OR = 1.56, 95% CI: 1.41–1.72) and Model 1 (OR = 1.29, 95% CI: 1.14–1.46), whereas later menopause was significantly associated with better cognition only in Model 1 (OR = 1.14, 95% CI: 1.00–1.29). Overall, these findings suggest that reproductive lifespan may serve as a more reliable indicator of late‐life cognitive health than the individual timing of menarche or menopause.

**TABLE 2:** Association between reproductive life span and cognitive function in women.

	Model 0		Model 1^a^		Model 2^b^	
	OR (95% CI)	*p*	OR (95% CI)	*p*	OR (95% CI)	*p*
Number	5586		4077		4077	
Short span	1 (Reference)	NA	1 (Reference)	NA	1 (Reference)	NA
Long span	1.40 (1.26,1.56)	<0.001^*^	1.31 (1.15,1.50)	<0.001^*^	1.20 (1.03,1.38)	0.017^*^
Number	6879		5157		5157	
Early menarche	1 (Reference)	NA	1 (Reference)	NA	1 (Reference)	NA
Late menarche	1.56 (1.41,1.72)	<0.001^*^	1.29 (1.14,1.46)	<0.001^*^	1.11 (0.98,1.27)	0.105
Number	6396		4776		4776	
Early menopause	1 (Reference)	NA	1 (Reference)	NA	1 (Reference)	NA
Late menopause	1.06 (0.96,1.17)	0.266	1.14 (1.00,1.29)	0.039^*^	1.07 (0.94,1.23)	0.306

*Note*: Model 0: Crude model (no adjustment).

Abbreviations: CI, confidence interval; OR, odds ratio; *p*, *p*‐value.

^a^Model 1: Adjusted for age and childhood cognitive proxies, including residential location, mother's education, father's education, childhood family economy, childhood family safety, and childhood physical health.

^b^Model 2: Further adjusted for adult education.

^*^= *p* < 0.05.

### 3.2 Number of children and cognition

#### 3.2.1 Characteristics of the study population

Table 3 presents the characteristics of all female participants, stratified by number of children. The mean age of the sample was 60.2 ± 9.2 years. Of the 7746 women 45 years of age or older, 4311 (55.7%) reported having one or two biological children, and 3435 (44.3%) reported having three or more. Women with more children exhibited significantly lower cognitive function than those with fewer children: 2317 (67.5%) in the more children group were classified as having low cognition, compared to 1886 (43.7%) in the fewer children group. Furthermore, women with more children had notably lower educational attainment (lower secondary: 10.7% vs 25.6%; upper secondary or above: 3.7% vs 14.2%). Among postmenopausal women, those with more children were also more likely to have a shorter reproductive lifespan (50.6% vs 45.5%) and earlier menarche (65.6% vs 54.3%). In addition, women with more children tended to be born earlier, had parents with lower levels of education, and reported slightly poorer childhood family economic conditions and greater childhood family insecurity. They were also less likely to be currently married, more likely to reside in rural areas, and exhibited somewhat higher rates of smoking and alcohol use compared with women with fewer children.

**TABLE 3:** Female participant characteristics by number of children in the China Health and Retirement Longitudinal Study, 2011–2018.

Characteristic	Overall *n* = 7746	fewer children *n* = 4311	more children *n* = 3435	*p* value
Age, years, mean (SD)	60.2 (9.2)	56.4 (7.2)	64.9 (9.3)	<0.001^*^
Education, *n* (%)				<0.001^*^
Preprimary	2177 (28.1)	791 (18.3)	1386 (40.3)	
Primary	3362 (43.4)	1806 (41.9)	1556 (45.3)	
Lower secondary	1469 (19.0)	1103 (25.6)	366 (10.7)	
Upper secondary or above	738 (9.5)	611 (14.2)	127 (3.7)	
Residence, *n* (%)				<0.001^*^
Urban	1482 (19.6)	1036 (24.6)	446 (13.3)	
Intergration zone	506 (6.7)	354 (8.4)	152 (4.5)	
Rural	5570 (73.7)	2820 (66.9)	2750 (82.1)	
Special zone	3 (< 1)	3 (0.1)	0 (0.0)	
Mother education, *n* (%)				<0.001^*^
Preprimary	6320 (85.0)	3265 (78.8)	3055 (92.9)	
Primary	922 (12.4)	715 (17.2)	207 (6.3)	
Lower secondary	124 (1.7)	106 (2.6)	18 (0.5)	
Upper secondary or above	66 (0.9)	59 (1.4)	7 (0.2)	
Father education, *n* (%)				<0.001^*^
Preprimary	3790 (53.7)	1869 (47.0)	1921 (62.2)	
Primary	2499 (35.4)	1531 (38.5)	968 (31.3)	
Lower secondary	460 (6.5)	333 (8.4)	127 (4.1)	
Upper secondary or above	315 (4.5)	241 (6.1)	74 (2.4)	
Childhood economy, *n* (%)				<0.001^*^
Better	865 (11.3)	518 (12.1)	347 (10.2)	
Same	4010 (52.2)	2304 (53.8)	1706 (50.2)	
Worse	2812 (36.6)	1464 (34.2)	1348 (39.6)	
Childhood safe, *n* (%)				<0.001^*^
Very safe	3720 (49.8)	2034 (48.7)	1686 (51.2)	
Somewhat safe	3116 (41.7)	1819 (43.5)	1297 (39.4)	
Not safe	637 (8.5)	326 (7.8)	311 (9.4)	
Childhood health, *n* (%)				0.018^*^
Healthier	2720 (35.4)	1574 (36.7)	1146 (33.8)	
Average	3931 (51.2)	2137 (49.9)	1794 (52.9)	
Less healthy	1025 (13.4)	575 (13.4)	450 (13.3)	
Marital Status, *n* (%)				<0.001^*^
Married	6330 (81.8)	3825 (88.9)	2505 (73.0)	
Others	1406 (18.2)	478 (11.1)	928 (27.0)	
Smoke, *n* (%)				<0.001^*^
Never smoke	6463 (93.2)	3707 (94.5)	2756 (91.6)	
Former smoke	156 (2.3)	67 (1.7)	89 (3.0)	
Current smoke	312 (4.5)	148 (3.8)	164 (5.5)	
Drink, *n* (%)				<0.001^*^
Never drink	5825 (75.2)	3249 (75.4)	2576 (75.0)	
Former drink	541 (7.0)	241 (5.6)	300 (8.7)	
Current drink	1379 (17.8)	821 (19.0)	558 (16.2)	
Disease, *n* (%)				<0.001^*^
Yes	5275 (68.4)	2997 (70.0)	2278 (66.4)	
No	2440 (31.6)	1287 (30.0)	1153 (33.6)	
Menarche age	16.0 (2.1)	15.7 (2.0)	16.3 (2.1)	<0.001^*^
Menarche age, *n* (%)				<0.001^*^
Early menarche	3606 (59.6)	1757 (54.3)	1849 (65.6)	
Late menarche	2448 (40.4)	1480 (45.7)	968 (34.4)	
Menopause age	49.3 (3.7)	49.4 (3.5)	49.3 (3.9)	0.13^*^
Menopause age, *n* (%)				0.20
Early menopause	2484 (44.6)	1299 (45.4)	1185 (43.7)	
Late menopause	3087 (55.4)	1561 (54.6)	1526 (56.3)	
Reproductive life span	33.4 (4.1)	33.6 (3.8)	33.1 (4.3)	<0.001^*^
Reproductive lifespan, *n*(%)				<0.001^*^
Short span	2284 (48.0)	1083 (45.5)	1201 (50.6)	
Long span	2471 (52.0)	1298 (54.5)	1173 (49.4)	
Children	2.7 (1.4)	1.7 (0.5)	3.9 (1.2)	<0.001^*^
Age at first live birth	24.6 (4.1)	24.7 (3.6)	24.5 (4.7)	0.068
Age at first live birth, *n* (%)				0.014^*^
Early age at first Birth	3336 (55.4)	1846 (54.0)	1490 (57.2)	
Late age at first birth	2684 (44.6)	1570 (46.0)	1114 (42.8)	
Cognitive z‐score	−0.2 (1.1)	0.1 (1.0)	−0.5 (1.0)	<0.001^*^
Cognitive function, *n* (%)				<0.001^*^
Low	4203 (54.3)	1886 (43.7)	2317 (67.5)	
High	3543 (45.7)	2425 (56.3)	1118 (32.5)	

* *p* < 0.05.

#### 3.2.2 Association between number of children and cognitive function

Table 4 summarizes the associations between number of children and cognitive function among middle‐aged and older women and men. In both sexes, having more children was significantly associated with a lower odds of high cognitive function compared to having fewer children. This association was more pronounced in women (OR = 0.38, 95% CI: 0.34–0.41) than in men (OR = 0.52, 95% CI: 0.47–0.57). This pattern persisted after adjustment for childhood cognitive proxies, remaining more pronounced in women. After further adjustment for adult education (Model 2), the association remained significant in women (OR = 0.87, 95% CI: 0.77–0.99), whereas in men the association was attenuated and no longer reached statistical significance (OR = 0.90, 95% CI: 0.80–1.01). The negative association between number of children and late‐life cognition was generally robust in women across other two adjusted models (Model 3: OR = 0.86, 95% CI: 0.76–0.99; Model 4: OR = 0.79, 95% CI: 0.70–0.89; see Table S3), and was further supported by multiple adjusted linear regression analyses (Table S2). Stratified analyses by educational level indicated that this negative association persisted across all educational strata, with the effect being more pronounced among women with higher education (OR decreased from 0.64 in preprimary to 0.47 in upper secondary or above; see Table S4). Regarding covariates, higher educational attainment was consistently associated with an increased odds of high cognitive function in both women and men. Among women, several additional factors—including older age, rural residence, unmarried status, poorer childhood family safety, and poorer childhood physical health—were significantly associated with lower cognitive function in Model 3 (see Table S5). In addition, later age at first live birth was associated with a higher odds of high cognitive function in the unadjusted model (OR = 1.14, 95% CI: 1.03–1.25); however, this association became non‐significant after sequential adjustment for covariates.

**TABLE 4:** Association between children number and age at first live birth with cognitive function in males and females (age at first live birth in females only).

	Model 0		Model 1^a^		Model 2^b^	
	OR (95% CI)	*p*	OR (95% CI)	*p*	OR (95% CI)	*p*
**Male participants**						
Number	7872		6934		6933	
Fewer children	1 (Reference)	NA	1 (Reference)	NA	1 (Reference)	NA
More children	0.52 (0.47,0.57)	<0.001^*^	0.84 (0.75,0.94)	0.003^*^	0.90 (0.80,1.01)	0.083
**Female participants**						
Number	7746		6568		6568	
Fewer children	1 (Reference)	NA	1 (Reference)	NA	1 (Reference)	NA
More children	0.38 (0.34,0.41)	<0.001^*^	0.70 (0.62,0.79)	<0.001^*^	0.87 (0.77,0.99)	0.034^*^
Number	6478		5186		5186	
Early age at first live birth753	1 (Reference)	NA	1 (Reference)	NA	1 (Reference)	NA
Late age at first live birth	1.14 (1.03,1.25)	0.011^*^	1.12 (0.99,1.26)	0.069	1.01 (0.89,1.15)	0.873^*^

Abbreviations: CI: confidence interval; OR, odds ratio; *p*, *p*‐value.

Model 0: Crude model (no adjustment).

^a^Model 1: Adjusted for age and childhood cognitive proxies, including residential location, mother's education, father's education, childhood family economy, childhood family safety, and childhood physical health.

^b^Model 2: Further adjusted for adult education.

* = *p* < 0.05.

## 4 DISCUSSION

This study is the first to concurrently examine the associations between reproductive factors and cognitive performance in middle‐aged and older women from both physiological and sociocultural perspectives. Drawing on nationally representative longitudinal data from China, we found that a longer reproductive lifespan was positively associated with better cognitive outcomes in postmenopausal women. In contrast, a higher number of biological children was associated with an elevated risk of cognitive impairment, with this adverse association significantly more pronounced in women than in men. These findings provide novel evidence on the cognitive implications of women's reproductive history and underscore the importance of integrating biological and social reproductive factors into dementia risk assessment and prevention strategies.

Accumulating evidence from large‐scale cohorts and meta‐analyses supports a robust association between female reproductive factors and later‐life cognitive health. Reproductive lifespan—an indicator of cumulative estrogen exposure—has been linked consistently to better cognitive outcomes. Studies from the United States and Singapore report that shorter reproductive spans are associated with an increased risk of cognitive impairment.^25^, ^26^ Meta‐analyses involving millions of women confirm that shorter reproductive span elevates the risk of dementia and cognitive decline.^13^, ^27^ Later menopause, reflecting a longer reproductive lifespan, is consistently associated with better cognition, whereas earlier menopause increases dementia risk across North America,^25^ Europe,^28^ and Asia.^29^ These associations are further supported by pooled analyses from multinational prospective studies.^13^, ^27^, ^30^ Evidence regarding age at menarche and later‐life cognition is more mixed. Some studies suggest that later menarche increases dementia risk,^13^, ^25^, ^28^, ^31^ whereas others—including findings from the UK Biobank—report the opposite.^32^ A dose–response meta‐analysis indicated a J‐shaped association,^27^ and several studies found no significant link.^33^, ^34^ Confounding by early‐life adversity and lower socioeconomic status—both associated with earlier menarche—may obscure underlying biological effects. Earlier menarche is also linked to younger maternal age at first birth, which has been associated with poorer cognitive outcomes in later life. Estrogen appears neuroprotective across both direct and indirect measures. Higher postmenopausal estradiol levels are inversely associated with Alzheimer's risk,^27^ and hormonal contraceptive use^15^, ^26^, ^28^, ^31^, ^32^ is linked to slower cognitive decline. However, the cognitive effects of hormone therapy (HT) remain debated, with benefits potentially dependent on formulation, dosage, and especially timing of initiation, ideally within 5 years of menopause.^15^, ^26^, ^28^, ^31^, ^32^ Neuroimaging studies further demonstrate that premature ovarian insufficiency is linked to structural and functional brain alterations, such as reduced gray matter volume,^35^ increased white matter hyperintensities,^35^ impaired synaptic integrity,^36^ and accelerated prefrontal cortical aging.^37^ Our findings from a Chinese cohort confirm that a longer reproductive lifespan is associated with better late‐life cognition in women, supporting a neuroprotective role of prolonged estrogen exposure. Although later menopause and later menarche were also linked to better cognition in some covariate‐adjusted models, these associations appear less robust compared with reproductive lifespan.

Much research has focused on the biological aspects of ovarian aging; however, this study also underscores the cognitive relevance of socially acquired reproductive experiences, particularly parity. Across diverse populations, having a higher number of children has been associated consistently with poorer cognitive outcomes among women. Studies from the United States,^15^ Latin America,^28^ Singapore,^26^ and Korea,^31^ as well as pooled data from 11 international cohorts,^38^ report elevated risks of cognitive decline or dementia with an increasing number of births. Notably, evidence from the UK Biobank shows that this association is sex specific: while fatherhood is linked to better cognitive performance in men, motherhood is associated with worse outcomes in memory and fluid intelligence.^39^ However, data from low‐ and middle‐income countries remain scarce. Using nationally representative CHARLS data, our study expands this literature by including both men and women ≥45 years of age. We found that having three or more children was significantly associated with lower cognitive performance, and that this negative effect was more pronounced in women than in men, independent of sociodemographic and health factors. These findings underscore the need to consider gender‐specific social exposures in cognitive aging research. It is important to note that having children versus being childless, and, among parents, the number of children, reflect conceptually distinct research questions. Since our study focuses on the effect of the number of children rather than the presence versus absence of children, participants without children (≈5%) were excluded from analyses of the number of children. In addition, although China's one‐child policy (1980–2015) may have influenced fertility patterns, most women in this cohort had completed childbearing before strict enforcement, and only 18.1% had a single child, suggesting a limited impact on the observed associations between number of children and late‐life cognitive function.

Several pathways may mediate the inverse association between number of children and late‐life cognition, spanning psychosocial, physiological, and sociocultural domains.^40^ Psychosocially, childbearing can constrain women's educational and occupational trajectories, as fertility typically declines after the mid‐30s, whereas most first births occur earlier,^41^ forcing trade‐offs between family and career. This contributes to lower fertility intentions among highly educated women globally.^42^ Motherhood is also associated with depressive symptoms and sleep disruption—the former encompassing postpartum depression,^43^, ^44^ disproportionate burdens of unpaid domestic labor,^45^, ^46^ and the psychological stress of childrearing,^47^ and the latter including pregnancy‐related and parenting‐related sleep deprivation.^48^, ^49^ These two domains reinforce one another, and both are established risk factors for cognitive decline.^50^, ^51^ Physiological mechanisms may also contribute: adverse pregnancy outcomes, such as recurrent stillbirths,^52^, ^53^ miscarriages,^52^, ^53^ hypertensive disorders of pregnancy,^54^, ^55^, ^56^, ^57^, ^58^ and gestational diabetes^59^ have each been linked to poorer late‐life cognitive outcomes and increased dementia risk, with neuroimaging evidence showing brain changes resembling those in Alzheimer's disease.^60^ Finally, sociocultural influences are particularly salient in East Asia, where traditional norms around marriage and childbearing may limit women's educational and occupational opportunities, and workplace barriers and postpartum constraints hinder reentry into the labor force. These structural and cultural pressures can reduce cognitive stimulation, foster social isolation, and shape reproductive choices. For example, despite extensive policy adjustments, fertility rates in Korea continue to decline, reflecting the complex interplay of gender norms, employment constraints, and reproductive decisions.^61^

### 4.1 Strengths and limitations

A key strength of this study is that it represents the first population‐based investigation in China to simultaneously examine both biological and sociocultural dimensions of ovarian aging and their associations with cognitive function in middle‐aged and older women. By integrating physiological indicators with reproductive experiences, our findings provide a more nuanced understanding of the long‐term cognitive implications of women's reproductive history. Notably, this study is the first to demonstrate that both reproductive lifespan and number of children are consistently associated with late‐life cognition in women, whereas other reproductive factors showed less‐robust associations. These insights underscore the importance of supporting women in navigating reproductive decisions that balance both immediate and later‐life health outcomes. Future research should explore interventions to mitigate the cognitive burden of childbearing, ranging from biological strategies to extend reproductive health to social policies aimed at reducing caregiving demands and emotional strain disproportionately borne by women. Such efforts may promote more equitable reproductive choices and support healthy cognitive aging.

This study has several limitations. First, the absence of detailed data on ovarian or uterine surgeries precluded differentiation between natural and surgical menopause. The recorded number of participants reporting a history of ovarian, cervical, or endometrial cancer (*n* = 117) was likely underestimated due to stigma‐related underreporting in older adults. Future studies should improve data completeness in this area. Second, we lacked information on exogenous hormone use, which limited our ability to identify women who may have extended reproductive span through hormone therapy; however, its impact is likely minimal given the low use among Chinese women. Third, although variables such as education, depressive symptoms, and sleep disturbances could theoretically mediate the relationship between childbearing and late‐life cognition, limitations in temporal data precluded accurate mediation analysis. Specifically, education could not be examined as a mediator because most women discontinued schooling after childbearing (only 126 of 6229 women continued schooling after their first child), whereas depressive symptoms and sleep disturbances were measured only a few years (up to ≈7 years) prior to the 2018 cognitive evaluation. Fourth, due to the absence of standardized definitions for reproductive age thresholds, extreme values for reproductive lifespan were excluded, and reproductive lifespan was dichotomized at the median to capture overall trends. Future research should examine cognitive outcomes among women with more extreme reproductive histories and explore standardized cutoff values. Finally, an additional wave of CHARLS data from 2020, collected during the coronavirus disease 2019 (COVID‐19) pandemic, was released in late 2024. Given the pronounced rise in depression and anxiety during this period—factors closely linked to cognitive outcomes—we deemed it inappropriate to combine these data with our pre‐pandemic analyses. As we enter a period of sustained coexistence with COVID‐19, future studies will incorporate these data to refine and extend our findings.

## 5 CONCLUSION

Longer reproductive span was associated with better cognitive function in postmenopausal women, whereas a higher number of children was linked to poorer cognition in both sexes, particularly in women. These associations were robust and stronger than those for other reproductive factors. Together, they highlight the dual biological and social dimensions of reproductive history in cognitive aging and suggest that number of children may serve as a sex‐specific consideration in dementia prevention strategies.

## CONFLICT OF INTEREST STATEMENT

The authors declare no conflict of interest. Any author disclosures are available in the Supporting Information.

## CONSENT STATEMENT

All participants provided written informed consent.

## Supporting information



Supporting Information

Supporting Information

## References

[1] WHO . World health statistics 2024: monitoring health for the SDGs, Sustainable Development Goals. World Health Organization; License: CC BY‐NC‐SA 3.0 IGO. 2024;

[2] Rajan KB , Weuve J , Barnes LL , McAninch EA , Wilson RS , Evans DA . Population estimate of people with clinical Alzheimer's disease and mild cognitive impairment in the United States (2020‐2060). Alzheimers Dement. 2021;17(12):1966‐1975. doi:10.1002/alz.12362 34043283 PMC9013315

[3] Alzheimer’s Association . 2024 Alzheimer's disease facts and figures. Alzheimers Dement. 2024;20(5):3708‐3821. doi:10.1002/alz.13809 38689398 PMC11095490

[4] Jia L , Du Y , Chu L , et al. Prevalence, risk factors, and management of dementia and mild cognitive impairment in adults aged 60 years or older in China: a cross‐sectional study. Lancet Public Health. 2020;5(12):e661‐e671. doi:10.1016/s2468-2667(20)30185-7 33271079

[5] Yang K , Yang X , Yin P , Zhou M , Tang Y . Temporal trend and attributable risk factors of Alzheimer's disease and other dementias burden in China: findings from the global burden of disease study 2021. Alzheimers Dement. 2024;20(11):7871‐7884. doi:10.1002/alz.14254 39312279 PMC11567818

[6] Ministry of Civil Affairs of the People's Republic of China . Statistical Bulletin on the Development of Undertakings for the Aged in China (2023). 2024. Retrieved from http://www.mca.gov.cn/

[7] Lopez‐Lee C , Torres ERS , Carling G , Gan L . Mechanisms of sex differences in Alzheimer's disease. Neuron. 2024;112(8):1208‐1221. doi:10.1016/j.neuron.2024.01.024 38402606 PMC11076015

[8] Wedatilake Y , Myrstad C , Tom SE , Strand BH , Bergh S , Selbæk G . Female reproductive factors and risk of mild cognitive impairment and dementia: the HUNT study. J Prev Alzheimers Dis. 2024;11(4):1063‐1072. doi:10.14283/jpad.2024.46 39044518 PMC11937202

[9] Ding H , Li Y , Ang TFA , et al. Reproductive markers in Alzheimer's disease progression: the Framingham Heart Study. J Prev Alzheimers Dis. 2023;10(3):530‐535. doi:10.14283/jpad.2023.28 37357294 PMC13264159

[10] Nakanishi M , Yamasaki S , Stanyon D , et al. Associations among age at menopause, depressive symptoms, and cognitive function. Alzheimers Dement. 2025;21(4):e70063. doi:10.1002/alz.70063 40231645 PMC11997867

[11] Brann DW , Lu Y , Wang J , et al. Brain‐derived estrogen and neural function. Neurosci Biobehav Rev. 2022;132:793‐817. doi:10.1016/j.neubiorev.2021.11.014 34823913 PMC8816863

[12] Russell JK , Jones CK , Newhouse PA . The role of estrogen in brain and cognitive aging. Neurotherapeutics. 2019;16(3):649‐665. doi:10.1007/s13311-019-00766-9 31364065 PMC6694379

[13] Han SL , Liu DC , Tan CC , Tan L , Xu W . Male‐ and female‐specific reproductive risk factors across the lifespan for dementia or cognitive decline: a systematic review and meta‐analysis. BMC Med. 2023;21(1):457. doi:10.1186/s12916-023-03159-0 37996855 PMC10666320

[14] Guo M , Wu Y , Gross AL , Karvonen‐Gutierrez C , Kobayashi LC . Age at menopause and cognitive function and decline among middle‐aged and older women in the China Health and Retirement Longitudinal Study, 2011‐2018. Alzheimers Dement. 2025;21(2):e14580. doi:10.1002/alz.14580 39936226 PMC11815216

[15] Lee JK , Frank RD , Christenson LR , Fields JA , Rocca WA , Mielke MM . Associations of reproductive factors and exogenous estrogens with global and domain‐specific cognition in later life. Alzheimers Dement. 2024;20(1):63‐73. doi:10.1002/alz.13394 37450421 PMC10787812

[16] Du Y , Luo Y , Zheng X , Liu J . Number of children and cognitive function among Chinese menopausal women: the mediating role of depressive symptoms and social participation. J Affect Disord. 2023;340:758‐765. doi:10.1016/j.jad.2023.08.084 37591349

[17] Song Y , Rosano C , Cvejkus RK , et al. Reproductive health history and later life cognition in African Caribbean women. Alzheimers Dement. 2025;21(8):e70547. doi:10.1002/alz.70547 40772453 PMC12329571

[18] Zhao Y , Hu Y , Smith JP , Strauss J , Yang G . Cohort profile: the China Health and Retirement Longitudinal Study (CHARLS). Int J Epidemiol. 2014;43(1):61‐68. doi:10.1093/ije/dys203 23243115 PMC3937970

[19] Zhao YJS , Chen X , Wang Y , et al. China Health and Retirement Longitudinal Study Wave 4 User's Guide, National School of Development. Peking University; 2020.

[20] OECD EU , UNESCO Institute for Statistics. ISCED 2011 Operational Manual: Guidelines for Classifying National Education Programmes and Related Qualifications, OECD Publishing. 2015. doi:10.1787/9789264228368-en

[21] Okoth K , Chandan JS , Marshall T , et al. Association between the reproductive health of young women and cardiovascular disease in later life: umbrella review. BMJ (Clinical research ed). 2020;371:m3502. doi:10.1136/bmj.m3502 PMC7537472 33028606

[22] Nichols AR , Chavarro JE , Oken E . Reproductive risk factors across the female lifecourse and later metabolic health. Cell Metab. 2024;36(2):240‐262. doi:10.1016/j.cmet.2024.01.002 38280383 PMC10871592

[23] Jin Y , Liang J , Hong C , Liang R , Luo Y . Cardiometabolic multimorbidity, lifestyle behaviours, and cognitive function: a multicohort study. Lancet Healthy Longev. 2023;4(6):e265‐e273. doi:10.1016/s2666-7568(23)00054-5 37150183

[24] Weaver NA , Kuijf HJ , Aben HP , et al. Strategic infarct locations for post‐stroke cognitive impairment: a pooled analysis of individual patient data from 12 acute ischaemic stroke cohorts. Lancet Neurol. 2021;20(6):448‐459. doi:10.1016/s1474-4422(21)00060-0 33901427

[25] Gilsanz P , Lee C , Corrada MM , Kawas CH , Quesenberry CP, Jr , Whitmer RA . Reproductive period and risk of dementia in a diverse cohort of health care members. Neurology. 2019;92(17):e2005‐e2014. doi:10.1212/wnl.0000000000007326 30923235 PMC6511081

[26] Song X , Wu J , Zhou Y , et al. Reproductive and hormonal factors and risk of cognitive impairment among Singapore Chinese women. Am J Obstet Gynecol. 2020;223(3):410. doi:10.1016/j.ajog.2020.02.032. e1‐e23. PMC7483640 32112728

[27] Fu C , Hao W , Shrestha N , Virani SS , Mishra SR , Zhu D . Association of reproductive factors with dementia: a systematic review and dose‐response meta‐analyses of observational studies. EClinicalMedicine. 2022;43:101236. doi:10.1016/j.eclinm.2021.101236 34977513 PMC8683685

[28] Stickel AM , Tarraf W , Kuwayama S , et al. Connections between reproductive health and cognitive aging among women enrolled in the HCHS/SOL and SOL‐INCA. Alzheimers Dement. 2024;20(3):1944‐1957. doi:10.1002/alz.13575 38160447 PMC10947951

[29] Yoo JE , Yoon DH , Jin EH , et al. Association between depression and young‐onset dementia in middle‐aged women. Alzheimers Res Ther. 2024;16(1):137. doi:10.1186/s13195-024-01475-y 38926887 PMC11201295

[30] Dobson AJ , Xu Z , Wilson LF , et al. Menopause age and type and dementia risk: a pooled analysis of 233 802 women. Age Ageing. 2024;53(11):afae254. doi:10.1093/ageing/afae254 39562342 PMC11576136

[31] Yoo JE , Shin DW , Han K , et al. Female reproductive factors and the risk of dementia: a nationwide cohort study. Eur J Neurol. 2020;27(8):1448‐1458. doi:10.1111/ene.14315 32396982

[32] Gong J , Harris K , Peters SAE , Woodward M . Reproductive factors and the risk of incident dementia: a cohort study of UK Biobank participants. PLoS Med. 2022;19(4):e1003955. doi:10.1371/journal.pmed.1003955 35381014 PMC8982865

[33] Najar J , Östling S , Waern M , et al. Reproductive period and dementia: a 44‐year longitudinal population study of Swedish women. Alzheimers Dement. 2020;16(8):1153‐1163. doi:10.1002/alz.12118 32573980

[34] Prince MJ , Acosta D , Guerra M , et al. Reproductive period, endogenous estrogen exposure and dementia incidence among women in Latin America and China; A 10/66 population‐based cohort study. PLoS One. 2018;13(2):e0192889. doi:10.1371/journal.pone.0192889 29489847 PMC5831083

[35] Liao H , Cheng J , Pan D , et al. Association of earlier age at menopause with risk of incident dementia, brain structural indices and the potential mediators: a prospective community‐based cohort study. EClinicalMedicine. 2023;60:102033. doi:10.1016/j.eclinm.2023.102033 37396803 PMC10314163

[36] Wood Alexander M , Honer WG , Saloner R , et al. The interplay between age at menopause and synaptic integrity on Alzheimer's disease risk in women. Sci Adv. 2025;11(10):eadt0757. doi:10.1126/sciadv.adt0757 40043118 PMC11881898

[37] Gunter‐Rahman F , Adams CD , Raju RM , Zhang Y , Lee EA , Messerlian C . Multiomic profiling reveals timing of menopause predicts prefrontal cortex aging and cognitive function. Aging Cell. 2025;24(2):e14395. doi:10.1111/acel.14395 39501567 PMC11822667

[38] Bae JB , Lipnicki DM , Han JW , et al. Does parity matter in women's risk of dementia? A COSMIC collaboration cohort study. BMC Med. 2020;18(1):210. doi:10.1186/s12916-020-01671-1 32753059 PMC7406389

[39] Zhang Y , Fletcher J , Lu Q , Song J . Gender differences in the association between parity and cognitive function: evidence from the UK biobank. Soc Sci Med. 2023;320:115649. doi:10.1016/j.socscimed.2022.115649 36709690 PMC9974636

[40] Dong L , Teh DBL , Kennedy BK , Huang Z . Unraveling female reproductive senescence to enhance healthy longevity. Cell Res. 2023;33(1):11‐29. doi:10.1038/s41422-022-00718-7 36588114 PMC9810745

[41] Deng K , Liang J , Mu Y , et al. Preterm births in China between 2012 and 2018: an observational study of more than 9 million women. Lancet Glob Health. 2021;9(9):e1226‐e1241. doi:10.1016/s2214-109x(21)00298-9 34416213 PMC8386289

[42] Yang S‐h , Zhang X‐l . Cohort differences in behaviors and concepts of marriage and childbearing among young women in China. Social Sciences of Beijing. 2023;10:95–106.

[43] World Health Organization . Guide for integration of perinatal mental health in maternal and child health services. World Health Organization; 2022. License: CC BY‐NC‐SA 3.0 IGO.

[44] Wang Z , Liu J , Shuai H , et al. Mapping global prevalence of depression among postpartum women. Transl Psychiatry. 2021;11(1):543. doi:10.1038/s41398-021-01663-6 34671011 PMC8528847

[45] Yavorsky JE , Dush CM , Schoppe‐Sullivan SJ . The production of inequality: the gender division of labor across the transition to parenthood. J Marriage Fam. 2015;77(3):662‐679. doi:10.1111/jomf.12189 26430282 PMC4584401

[46] Nomaguchi K , Milkie MA . Parenthood and well‐being: a decade in review. J Marriage Fam. 2020;82(1):198‐223. doi:10.1111/jomf.12646 32606480 PMC7326370

[47] Ruppanner L , Perales F , Baxter J . Harried and unhealthy? parenthood, time pressure, and mental health. J Marriage Fam. 2019;81(2):308‐326. doi:10.1111/jomf.12531

[48] Rajagopal A , Sigua NL . Women and sleep. Am J Respir Crit Care Med. 2018;197(11):P19‐p20. doi:10.1164/rccm.19711P19 29856255

[49] Pengo MF , Won CH , Bourjeily G . Sleep in women across the life span. Chest. 2018;154(1):196‐206. doi:10.1016/j.chest.2018.04.005 29679598 PMC6045782

[50] Semkovska M , Quinlivan L , O'Grady T , et al. Cognitive function following a major depressive episode: a systematic review and meta‐analysis. Lancet Psychiatry. 2019;6(10):851‐861. doi:10.1016/s2215-0366(19)30291-3 31422920

[51] Irwin MR , Vitiello MV . Implications of sleep disturbance and inflammation for Alzheimer's disease dementia. Lancet Neurol. 2019;18(3):296‐306. doi:10.1016/s1474-4422(18)30450-2 30661858

[52] Liang C , Dobson AJ , Chung HF , et al. Association of infertility and recurrent pregnancy loss with the risk of dementia. Eur J Epidemiol. 2024;39(7):785‐793. doi:10.1007/s10654-024-01135-3 38888679 PMC11343804

[53] Miller EC , Conley P , Alirezaei M , et al. Associations between adverse pregnancy outcomes and cognitive impairment and dementia: a systematic review and meta‐analysis. Lancet Healthy Longev. 2024;5(12):100660. doi:10.1016/j.lanhl.2024.100660 39675366 PMC11726346

[54] Mielke MM , Frank RD , Christenson LR , Fields JA , Rocca WA , Garovic VD . Association of Hypertensive Disorders of Pregnancy With Cognition in Later Life. Neurology. 2023;100(19):e2017‐e2026. doi:10.1212/wnl.0000000000207134 36859405 PMC10186223

[55] Alers RJ , Ghossein‐Doha C , Canjels LPW , et al. Attenuated cognitive functioning decades after preeclampsia. Am J Obstet Gynecol. 2023;229(3):294. e1‐e14. doi:10.1016/j.ajog.2023.02.020 36863645

[56] Schliep KC , McLean H , Yan B , et al. Association between hypertensive disorders of pregnancy and dementia: a systematic review and meta‐analysis. Hypertension (Dallas, Tex : 1979). 2023;80(2):257‐267. doi:10.1161/hypertensionaha.122.19399 36345823 PMC9851987

[57] Prodan CI . Bridging the gap between hypertensive disorders of pregnancy and cognitive decline in older women. Neurology. 2023;100(19):893‐894. doi:10.1212/wnl.0000000000207237 36859409

[58] Carey C , Mulcahy E , McCarthy FP , et al. Hypertensive disorders of pregnancy and the risk of maternal dementia: a systematic review and meta‐analysis. Am J Obstet Gynecol. 2024;231(2):196‐210. doi:10.1016/j.ajog.2024.01.013 38278201

[59] Soria‐Contreras DC , Wang S , Liu J , et al. Lifetime history of gestational diabetes and cognitive function in parous women in midlife. Diabetologia. 2025;68(1):105‐115. doi:10.1007/s00125-024-06270-w 39240352 PMC11960863

[60] Ijomone OK , Shallie P , Naicker T . Changes in the structure and function of the brain years after Pre‐eclampsia. Ageing Res Rev. 2018;47:49‐54. doi:10.1016/j.arr.2018.06.006 30026172

[61] Choi S , Ham S , Yang Y , Pareliussen J . Women’s employment and fertility in Korea: A literature review. OECD Economics Department Working Papers. No. 1825. OECD Publishing; 2024. Retrieved from https://www.oecd.org/en/publications/women‐s‐employment‐and‐fertility‐in‐korea_ac53879e‐en.html

