# Supplementary material for: Biological and social reproductive factors and late‐life cognitive function in middle‐aged and older Chinese women
Source: Alzheimers Dement. 2025 Oct 22;21(10):e70824. doi: 10.1002/alz.70824 (PMC12541285; doi:10.1002/alz.70824)
Supplement: Supplementary file 2 — Supporting Information [file ALZ-21-e70824-s002.docx]

**Supplementary Methods**

**Covariates**：

(1) Age at cognitive assessment was derived from the same survey wave as the cognitive outcome.
(2) Relatively stable or current status variables (residence, adult education, age at education completion, marital status, drinking status, smoking status, and physical diseases) were obtained from the most recent wave (2018). When missing, data were supplemented sequentially from 2015, 2013, and 2011.
(3) Childhood cognitive proxy variables (mother’s education, father’s education, childhood family economy, childhood family safety, and childhood physical health) were collected exclusively from the 2014 Life History Survey.

**Exposures**：

(1) Reproductive lifespan (= age at menopause – age at menarche) was obtained from fixed questions asked across the 2011–2018 waves. We prioritized 2011 data, supplemented by subsequent waves where missing. When direct age values were unavailable, age was calculated using the reported calendar year of menarche/menopause minus birth year, to maximize data coverage.

(2) Number of children was obtained from the 2014 Life History Survey.

(3) Age at first birth was calculated as the difference between the participant’s birth year and the birth year of the first child(2018 wave preferred, supplemented by 2015, 2013, or 2011 if missing).

**Outcomes：**

Cognitive outcomes were derived from tasks integrated within the same survey wave, prioritizing 2018, and supplemented sequentially by 2015, 2013, and 2011 if missing. To ensure correct temporal ordering, we restricted the analytic sample to cases where cognitive assessments occurred after menopause.

**Supplementary Tables**

# STable 1. Association of reproductive lifespan with cognitive function in women (Models 3 and 4)

|  | **Model 3^c^** | | **Model 4^d^** | |
| --- | --- | --- | --- | --- |
|  | **OR(95%CI)** | **P** | **OR(95%CI)** | **P** |
| Number | 3,751 |  | 4,753 |  |
| short span | 1 (Reference) | NA | 1 (Reference) | NA |
| long span | 1.15 (0.99,1.34) | 0.077 | 1.20 (1.05,1.37) | 0.006* |
| Number | 4,709 |  | 5,844 |  |
| early menarche | 1 (Reference) | NA | 1 (Reference) | NA |
| late menarche | 1.10 (0.96,1.26) | 0.174 | 1.10 (0.97,1.24) | 0.139 |
| Number | 4,419 |  | 5,519 |  |
| early menopause | 1 (Reference) | NA | 1 (Reference) | NA |
| late menopause | 1.05 (0.91,1.21) | 0.494 | 1.05 (0.93,1.19) | 0.416 |

Note: Model 3^c^: Adjusted for age and childhood cognitive proxies, adult education, marital status, drinking status, smoking status, and physical diseases.

Model 4^d^: Adjusted for age, adult education, marital status, drinking status, smoking status, and physical diseases.

*=p < 0.05

Abbreviations: OR: odds ratio; CI: confidence interval; P: p-value.

# STable 2. Associations of reproductive lifespan and number of children with cognitive outcomes in women: A Linear Regression Analysis

|  | **Reproductive lifespan** | | | **Children** | | |
| --- | --- | --- | --- | --- | --- | --- |
|  | **Estimate** | **SE** | **P** | **Estimate** | **SE** | **P** |
| Model 0 | 0.029 | 0.003 | <0.001* | -0.254 | 0.008 | <0.001* |
| Model 1 | 0.013 | 0.004 | <0.001* | -0.097 | 0.010 | <0.001* |
| Model 2 | 0.004 | 0.003 | 0.275 | -0.039 | 0.010 | <0.001* |
| Model 3 | 0.002 | 0.003 | 0.571 | -0.036 | 0.010 | <0.001* |
| Model 4 | 0.010 | 0.003 | 0.002* | -0.069 | 0.009 | <0.001* |

Note: Model 0: Crude model (no adjustment). Reproductive lifespan: n=5,586; Children: n=7,746.

Model 1^a^: Adjusted for age and childhood cognitive proxies, including residential location, mother’s education, father’s education, childhood family economy, childhood family safety, and childhood physical health. Reproductive lifespan: n=4,077; Children: n=6,568.

Model 2^b^: Further adjusted for adult education. Reproductive lifespan: n=3,965; Children: n=6,368

Model 3^c^: Further adjusted for marital status, drinking status, smoking status, and physical diseases. Reproductive lifespan: n=3,751; Children: n=6,037.

Model 4^d^: Adjusted for age, adult education, marital status, drinking status, smoking status, and physical diseases. Reproductive lifespan: n=4,753; Children: n=6,931.

*=p < 0.05

Abbreviations: SE: standard error; P: p-value.

# **STable 3. Association of** children number and age at first live birth **with cognitive function in men and women (Models 3 and 4)**

|  | **Model 3^c^** | | **Model 4^d^** | |
| --- | --- | --- | --- | --- |
|  | **OR (95%CI)** | **P** | **OR (95%CI)** | **P** |
| **Male participants** |  |  |  |  |
| Number | 5,755 |  | 6,345 |  |
| fewer children | 1 (Reference) | NA | 1 (Reference) | NA |
| more children | 0.92 (1.81,1.05) | 0.216 | 0.85 (0.75,0.95) | 0.006* |
| **Female participants** |  |  |  |  |
| Number | 6,037 |  | 6,931 |  |
| fewer children | 1 (Reference) | NA | 1 (Reference) | NA |
| more children | 0.86 (0.76,0.99) | 0.033* | 0.79 (0.70,0.89) | <0.001* |
| Number | 4,659 |  | 5,820 |  |
| early age at first live birth | 1 (Reference) | NA | 1 (Reference) | NA |
| late age at first live birth | 0.99 (0.87,1.13) | 0.883 | 1.02 (0.90,1.15) | 0.730 |

Note: Model 3^c^: Adjusted for age and childhood cognitive proxies, adult education, marital status, drinking status, smoking status, and physical diseases.

Model 4^d^: Adjusted for age, adult education, marital status, drinking status, smoking status, and physical diseases.

*=p < 0.05

Abbreviations: OR: odds ratio; CI: confidence interval; P: p-value.

# STable 4. Association of Number of Children with Cognitive Function Stratified by Educational Level in Women (Unadjusted Model).

Given the significant association between education and late-life cognition (STable 4) and the potential influence of education on childbearing reported in prior studies, we conducted stratified analyses by educational level. The results are presented below.

| **Education** | **Unadjusted Model** | |
| --- | --- | --- |
|  | **OR(95%CI)** | **P** |
| fewer children | 1 (Reference) | NA |
| preprimary (n=2,177) | 0.64 (0.50,0.81) | <0.001* |
| primary (n=3,362) | 0.62 (0.54,0.71) | 0.001* |
| lower secondary (n=1,469) | 0.60 (0.47,0.77) | <0.001* |
| upper secondary or above (n=738) | 0.47 (0.29,0.76) | 0.002* |

# STable 5. Covariate regression coefficients in model 3: association between number of children and cognitive function

| **Independent variable** | **male(n=5,755)** | | **female(n=6,037)** | |
| --- | --- | --- | --- | --- |
|  | **OR (95%CI)** | **P** | **OR (95%CI)** | **P** |
| Age | 0.97 (0.96–0.97) | <0.001* | 0.97 (0.96–0.98) | <0.001* |
| **Education** |  |  |  |  |
| preprimary | 1(reference) | NA | 1(reference) | NA |
| primary | 4.28 (3.21–5.71) | <0.001* | 4.58 (3.89–5.41) | <0.001* |
| lower secondary | 7.58 (5.61–10.23) | <0.001* | 9.65 (7.88–11.81) | <0.001* |
| upper secondary or above | 13.02 (9.42–18.01) | <0.001* | 20.38 (15.20–27.31) | <0.001* |
| **Residence** |  |  |  |  |
| urban | 1(reference) | NA | 1(reference) | NA |
| intergration zone | 0.92 (0.70–1.21) | 0.567 | 1.11 (0.84–1.45) | 0.467 |
| rural | 0.64 (0.54–0.76) | <0.001* | 0.57 (0.48–0.67) | <0.001* |
| **mother education** |  |  |  |  |
| preprimary | 1(reference) | NA | 1(reference) | NA |
| primary | 1.12 (0.92–1.37) | 0.258 | 1.11 (0.92–1.34) | 0.288 |
| lower secondary | 1.87 (1.04–3.36) | 0.037* | 1.53 (0.92–2.57) | 0.104 |
| upper secondary or above | 1.21 (0.59–2.51) | 0.605 | 1.59 (0.71–3.55) | 0.257 |
| **Father education** |  |  |  |  |
| preprimary | 1(reference) | NA | 1(reference) | NA |
| primary | 1.26 (1.11–1.43) | <0.001* | 1.08 (0.94–1.23) | 0.262 |
| lower secondary | 0.95 (0.70–1.28) | 0.738 | 1.09 (0.85–1.40) | 0.504 |
| upper secondary or above | 1.51 (1.07–2.13) | 0.020* | 1.59 (1.14–2.23) | 0.007* |
| **Childhood economy, n (%)** |  |  |  |  |
| better | 1(reference) | NA | 1(reference) | NA |
| same | 1.09 (0.88–1.36) | 0.428 | 0.94 (0.77–1.16) | 0.557 |
| worse | 1.05 (0.83–1.31) | 0.694 | 0.85 (0.69–1.06) | 0.147 |
| **Childhood safe, n (%)** |  |  |  |  |
| very safe | 1(reference) | NA | 1(reference) | NA |
| somewhat safe | 1.04 (0.92–1.17) | 0.506 | 1.20 (1.06–1.36) | 0.004* |
| not safe | 0.96 (0.77–1.20) | 0.720 | 0.77 (0.61–0.97) | 0.024* |
| **Childhood health** |  |  |  |  |
| healthier | 1 (Reference) | NA | 1 (Reference) | NA |
| average | 0.98 (0.87–1.11) | 0.791 | 0.81 (0.72–0.93) | 0.002* |
| less healthy | 0.89 (0.73–1.08) | 0.226 | 0.79 (0.65–0.97) | 0.021* |
| **Marital Status** |  |  |  |  |
| married | 1 (Reference) | NA | 1 (Reference) | NA |
| others | 0.82 (0.66–1.02) | 0.071 | 0.76 (0.64–0.91) | 0.003* |
| **Smoke, n (%)** |  |  |  |  |
| never smoke | 1 (Reference) | NA | 1(Reference) | NA |
| former smoke | 1.10 (0.93–1.30) | 0.272 | 0.99 (0.66–1.49) | 0.960 |
| current smoke | 0.71 (0.61–0.82) | <0.001* | 1.14(0.86–1.53) | 0.364 |
| **Drink, n (%)** |  |  |  |  |
| never drink | 1 (Reference) | NA | 1(Reference) | NA |
| former drink | 0.86 (0.72–1.04) | 0.122 | 0.87(0.68–1.12) | 0.286 |
| current drink | 1.11 (0.97–1.28) | 0.141 | 1.12(0.95–1.31) | 0.168 |
| **disease, n (%)** |  |  |  |  |
| yes | 1 (Reference) | NA | 1(Reference) | NA |
| no | 0.86 (0.76–0.98) | 0.019* | 1.03(0.90–1.17) | 0.677 |

Note: Model 3 was adjusted for age; childhood cognitive proxies (residential location, mother’s education, father’s education, childhood family economy, childhood family safety, and childhood physical health); adult education; marital status; drinking status; smoking status; and physical diseases.

*=p < 0.05.

Abbreviations: OR: odds ratio; CI: confidence interval; P: p-value.
